# Implementing a Public Health Objective for Alcohol Premises Licensing in Scotland: A Qualitative Study of Strategies, Values, and Perceptions of Evidence

**DOI:** 10.3390/ijerph14030221

**Published:** 2017-02-23

**Authors:** Niamh Fitzgerald, James Nicholls, Jo Winterbottom, Srinivasa Vittal Katikireddi

**Affiliations:** 1 Institute for Social Marketing, UK Centre for Tobacco and Alcohol Studies, Faculty of Health Sciences and Sport, University of Stirling, Stirling FK9 4LA, UK; 2 Alcohol Research UK, London SW1H 0HW, UK; James.nicholls@alcoholresearchuk.org; 3 Centre for History in Public Health, London School of Hygiene and Tropical Medicine, London WC1H 9SH, UK; 4 West Dunbartonshire Health and Social Care Partnership, Dumbarton G82 3PU, UK; jo.winterbottom@ggc.scot.nhs.uk; 5 MRC/CSO Social and Public Health Sciences Unit, University of Glasgow, Glasgow G2 3QB, UK; vittal.katikireddi@glasgow.ac.uk

**Keywords:** alcohol, licensing, outlet density, public involvement, availability

## Abstract

The public health objective for alcohol premises licensing, established in Scotland in 2005, is unique globally. We explored how public health practitioners engaged with the licensing system following this change, and what helped or hindered their efforts. Semi-structured interviews were conducted with 13 public health actors, audio-recorded, and analysed using an inductive framework approach. Many interviewees viewed the new objective as synonymous with reducing population-level alcohol consumption; however, this view was not always shared by licensing actors, some of whom did not accept public health as a legitimate goal of licensing, or prioritised economic development instead. Some interviewees were surprised that the public health evidence they presented to licensing boards did not result in their hoped-for outcomes; they reported that licensing officials did not always understand or value health data or statistical evidence. While some tried to give “impartial” advice to licensing boards, this was not always easy; others were clear that their role was one of “winning hearts and minds” through relationship-building with licensing actors over time. Notwithstanding the introduction of the public health objective, there remain significant, and political, challenges in orienting local premises licensing boards towards decisions to reduce the availability of alcohol in Scotland.

## 1. Introduction

Alcohol consumption is the leading cause of death amongst 15–49 year-olds worldwide [1] and is a major contributor to the preventable burden of disease in the UK and internationally [2,3]. There are over 1 million alcohol-related hospital admissions a year in England, and in 2013 there were 6592 alcohol-related deaths, a 10% increase from 2003 [4]. Alcohol consumption is also associated with adverse social outcomes like crime, job loss, and violence [5,6,7], causing a significant burden of harm to those other than the drinker. Furthermore, alcohol harms are socially patterned, making alcohol a key driver of health inequalities [8,9]. There is no definitive estimate of the economic cost of alcohol consumption, however, the most recent estimate for England and Wales is an annual cost of at least £21 billion [7,10]. Although there have been recent downward trends in harms, these have been from a very high level and may reverse if fiscal policy levers remain underused [11]. In Scotland, rates of harm have stabilized over recent years, with almost 35,000 alcohol-related hospitalizations in 2015–2016 [12]. After falls in sales between 2009 and 2013, alcohol sales in Scotland have increased again recently, and levels of consumption remain very high with an average of 10.7 L of pure alcohol per adult sold in 2015 (equating to 20.8 units per adult per week), 20% higher than in England/Wales [13].

Many public health experts argue that tackling alcohol-related harms requires action to reduce its affordability, marketing, and availability [14,15]. While there has been much public debate about the potential for minimum unit pricing of alcohol to address affordability in the UK [16,17], there has also been renewed interest in local approaches to the provision of premises licences for the sale of alcohol as a means to control availability [18,19,20,21]. There is consistent evidence suggesting an association between increased availability of alcohol, including the number and proximity of alcohol outlets in an area, and higher rates of consumption and associated alcohol-related harms [22,23,24,25], including some studies from Scotland [26,27]. However, the extent to which this association reflects a causal relationship and, if so, the mechanisms by which effects are exerted, remains the subject of study, since much of the research is cross-sectional and the validity of measures of the availability of alcohol premises uncertain [25,28,29,30,31,32]. 

Many countries, including the constituent countries of the UK, have restricted which premises are allowed to sell alcohol through the issuing of permits by local authorities (or local legislators in Northern Ireland) [21,25,31,33]. Historically, UK licensing systems developed as administrative and reactive systems originally designed as a means of limiting public disorder and regulating behaviour, though health considerations have played a part, albeit limited, in motivating legislative change [18,34]. Under reforms to the licensing systems in England and Wales (2003) and Scotland (2005) many discretionary aspects of licensing were formalised. The essential principle of these reforms is that alcohol licence applications can only be refused if (a) there is a formal representation from a “responsible authority” (e.g., the health board in Scotland or the police or fire service), and (b) that representation shows the application threatens to undermine one or more of the statutory “licensing objectives” (see Table 1). 

The introduction of a “fifth licensing objective” to “protect and improve public health” in the Licensing (Scotland) Act 2005 sets Scotland apart from the rest of the UK and is unique, globally, although some jurisdictions (including some Australian states and territories) have a requirement to consider “harm minimisation” in licensing decision-making [35]. The idea of making public health a formal consideration in licensing had first been raised at a 1997 conference convened by the then Scottish Office [36] (p. 18). From the late 1990s, health groups such as the Scottish Council on Alcohol Misuse and the Scottish Council on Alcohol (later Alcohol Focus Scotland)—working in an environment in which both licensing and public health were devolved to the new Scottish Government—pushed for a greater role for public health in regulating alcohol retail [37] (pp. 118–121). In 2003, the Nicholson Committee review of licensing, having established a health subgroup including a consultant physician, a director of public health and the director of the Central Scotland Council on Alcohol, recommended the introduction of a public health licensing objective [38]. This recommendation led directly to the inclusion of this provision in the 2005 Licensing Act.

The 2005 Act also stipulated that local licensing boards must produce a regular Statement of Licensing Policy, part of which must include a statement on “overprovision”; that is, whether there are areas in the board’s jurisdiction in which the number or density of outlets was excessive. It further empowered boards to, following consultation, define “overprovision areas” in which the assumption would be that any licence application be refused unless it could demonstrate that it did not threaten to undermine the licensing objectives. The Act does not provide a definitive description of what constitutes overprovision, but directs that Licensing Boards can have regard to the number, capacity, and type of premises in a given locality when setting out an overprovision statement [39]. Consultation prior to the establishment of an overprovision policy involves the local Chief Constable, local health board (including local public health departments of the National Health Service (NHS)), licence holders, residents and “such other persons as the [licensing] board sees fit” [39] (Section 7, paragraph 4). Importantly, boards have to identify an area as potentially overprovided prior to consultation, rather than inviting suggestions for possible overprovision areas through the consultation process [40]. A 2014 evaluation of the Licensing (Scotland) Act 2005 conducted predominantly with licensing board members and other licensing actors found that the new public health objective was viewed as “problematical” and that the concept of overprovision was felt to be difficult to define and measure [41]. 

The changes in the licensing regime were followed within three years by a national alcohol strategy in which the Scottish Government proposed, and then adopted, a “whole population” or public health focused approach to reducing alcohol-related harms [42,43]. Together, these developments led various public health stakeholders in Scotland to pay more attention to alcohol licensing as a policy venue for reducing alcohol-related harm. This study aimed to explore how these public health actors attempted to influence local alcohol licensing policies and decisions in Scotland towards the licensing objective of “protecting and improving public health”, and to identify factors felt to have helped or hindered their efforts. 

## 2. Materials and Methods

### 2.1. Sample

“Public health actors” were defined as individuals with a substantial remit to protect and promote public health generally or specifically in relation to alcohol, and included public health doctors in local NHS boards, or practitioners in various posts within alcohol and drug partnerships (strategic multi-agency planning and commissioning partnerships with a remit to reduce alcohol and illicit drug-related harms in a local area). Public health actors were eligible to take part in the study if they had recent and in-depth experience of engaging with local licensing boards, or trying to influence local licensing policy and decisions.

Eligible public health actors were identified in two ways: by reviewing publicly-available information describing prior efforts to protect public health through licensing; and via snowball sampling, principally via one key informant (already known to Niamh Fitzgerald)at Alcohol Focus Scotland (AFS), a voluntary sector organisation which has provided extensive support to local areas on this issue. Twelve local public health actors were identified in this way and approached to participate: 11 of these agreed to be interviewed; the twelfth individual declined to participate, citing a lack of action on this issue in her area as the reason. We also interviewed the key informant from AFS, along with another individual with a local authority licensing role recognised for long-standing and innovative work in this area. No further participants were sought: between them, the 13 interviewees covered almost all of the boards (20/22) which had declared any form of overprovision of licensed premises at the time, including 20 of the 40 licensing board areas in Scotland, as outlined in detail in Table 2 [44].

### 2.2. Data Collection

In-depth qualitative interviews were used to explore the complexity of processes by the legislation change led to changes in practice, insight and understanding at multiple levels. Qualitative research enables insight into people’s meanings and experiences and highlights social processes, which may be important in efforts to influence policy at this local level [45,46].

Interviewees were sent a study information sheet by email and followed up by telephone. Full informed consent was recorded prior to semi-structured interviews (averaging 69 min in duration) conducted by Niamh Fitzgerald between February and May 2014. Interviews were conducted mainly by telephone as we have previously found this to facilitate participation by professionals in similar roles [47]. Interviewees were also given the option of being interviewed face to face: just one (a key informant) chose to do so. This was after boards were expected to have published triennial statements of alcohol policy in November 2013. Interviewees were provided with a topic guide (summarised in Table 3) in advance, developed by Niamh Fitzgerald. 

During interviews, participants were encouraged to speak freely about their experiences: questions were not asked verbatim of each participant; the topic guide and issues raised by other interviewees were used as prompts. All interviews were audio-recorded: six were transcribed from the recordings after the interview; the other seven were simultaneously transcribed during the interviews. In both cases, the recordings were used afterwards to correct the transcripts. As a further check, all transcripts were subsequently sent to interviewees to check for accuracy at which point they also had the opportunity to elaborate or clarify any points as they saw fit. Emergent themes were not discussed with participants at this point.

### 2.3. Analysis

Analysis was thematic rather than theoretical. Notes and recordings were reviewed throughout the data collection period and full analysis was conducted afterwards using a framework approach as described by Gale et al. [48]. Niamh Fitzgerald and Jo Winterbottom independently coded two interviews manually, using open coding. They then met to discuss codes and broader themes arising and to agree on a draft coding framework. This was refined by both following analysis of three further interviews and then re-applied manually to all interviews by Niamh Fitzgerald. Formal inter-coder reliability checks were not conducted. A framework matrix was used to chart the data using Microsoft Excel (Microsoft Corporation, Redmond, WA, USA), enabling a holistic, descriptive overview of the entire data set to be taken. The themes of the study were then discussed in further detail with all authors to plan this paper.

### 2.4. Ethics

Ethical approval was granted by the Ethics Committee of the School of Management at the University of Stirling. When reviewing the interview transcripts for accuracy, interviewees were also invited to highlight any segments of interview which they felt might identify them, and agreement was reached as to how these would be used. For example, in some cases it was agreed that the interview identification number or the interviewee’s organisation type would not be used in conjunction with a specific quotation.

## 3. Results

Interviewees provided rich free-flowing descriptions of their experiences in seeking to orient local alcohol licensing towards a public health objective, outlining what they did and why, how others reacted, how they perceived the outcome of their efforts and what they had been surprised by or felt they had learned in the process. This paper focuses on how interviewees encountered differing values and beliefs about alcohol, alcohol premises licensing and evidence in general and how they viewed their role in engaging with the licensing process. These themes are reported in more detail below, drawing on several themes (2c Helping and Influencing, 2d Raising Awareness, 3c Conflicts of Interest, 4e Perceptions of Data, and 5 Attitudes and Beliefs Regarding Alcohol and Alcohol Licensing) from the overall analysis framework (summarised in Table 4). 

### 3.1. Values and Beliefs about Alcohol and Alcohol Licensing

Interviewees reported that the values and beliefs of all involved in licensing were important in determining the success of their endeavours, noting the need to “*take the temperature of the licensing board to guide what you do*” [L416, Interview 1, ADP]. They reported having to address what they felt were myths and stereotypes raised by members of licensing boards and licensing forums, such as a belief that alcohol problems related only to young people’s drinking, or to those who were dependent on alcohol. 

From the perspective of interviewees, the implementation of a public health objective for licensing required an acceptance of a “whole population approach” to alcohol policy, and the idea that alcohol consumption needed to fall across all groups. Many participants felt that this idea had not been fully accepted by others, and in fact, that there was a lack of consensus about alcohol problems.

“*We could endlessly explain the size of the problem for a particular area but action depends on having a general consensus that there is a problem but we don’t know where. It’s different if no-one is convinced that there’s even a problem to begin with.*”

[L357, Interview 1, ADP]

“*When we’re trying to make a case that there is too much alcohol being sold and drunk and its causing people long-term health harm, I don’t think as a society, people are generally in agreement with that necessarily…There’s a perspective that other people’s drinking may be harmful but my drinking is perfectly alright.*”

[L704, Interview 12, Public Health]

Participants tried to understand this perspective: one suggested that alcohol was a “*key feature common to every aspect of politics’,* that *‘alcohol lubricates political discussion, facilitates fundraisers*” [L372, Interview number and type withheld], and that because of this “*councillors are often torn between the academic perspective and their own experience.*” [L381].

Secondly, despite the public health objective, participants did not find a consensus that addressing public health was a legitimate role of licensing, one noting that “*[some licensing board members] will never believe in licensing having a health role and others…are passionate about [a health role].*” [L215, Interview 8, Key Informant]. Others noted that, in certain cases, licensing boards prioritised economic development over public health considerations.

“*Boards are asked to make decisions based on the five [*licensing*] objectives however the key element that dominates the agenda is the local economy rather than any discussions about the licensing objectives…It is all about profit and has very little impact on what the licensing board are supposed to do.*”

[L46, Interview 4, ADP]

“*[*In one area*], they still see attracting business to the town centre as important and they still seem to think that that business has to be associated with alcohol in one form or another which is a bit of a shame.*”

[L256, Interview 9, Public Health]

Some interviewees tried to counter economic arguments by presenting data on the harms of alcohol such as the cost of fires, or loss of productivity due to over-consumption, however they felt that “*a piece of work needs to be done in relation to whether more jobs equals better health*” [L49, Interview 6, ADP].

There were mixed views, both amongst interviewees, and in their reports of the views of others, on whether licensing could actually make a difference to public health. Some felt that it was only a small part of a bigger policy picture, but emphasised that it is “*the one that we have local control over*” [L352, Interview 13, Public Health]. Others noted that licensing could only stop increases rather than lead to decreases in premises numbers, and could do little about online sales for home drinking. For these reasons, one participant questioned whether all the effort on overprovision was “*worth the time invested*” [L688, Interview 12, Public Health]. 

### 3.2. Values and Beliefs about Evidence

One key informant reported his view that “*if the licensing board had the full data of the extent of alcohol problems in the area they generally would be horrified. They would think ‘we have to do something*’” [L353, Interview 8]. However a greater number of interviewees were surprised that the public health evidence they presented to licensing boards “*didn’t result in the outcome we were hoping [for]*” [L122, Interview 1, ADP].

“*The Licensing Board completely ignored the evidence. That let us see what an enormous task this is.*”

[L79, Interview 4, ADP]

“*An extensive range of evidence was provided and referred to…to demonstrate concerns about the health and social impact…No evidence was identified that objectively indicated a lack of availability. So we were pretty shocked that the Licensing Board had taken that position [of not declaring overprovision]…We were quite flabbergasted actually.*”

[L327, Interview 11, ADP]

Interviewees felt that councillors did not always have a good understanding of what constituted good evidence (as they saw it). One described how a councillor who was on the licensing forum would refer to trade magazines as evidence and another councillor “*declared*” that, in his view, there were a lot of places in a particular area which actually had no pub close to them and where the residents would welcome another pub. In this latter case, the interviewee noted that “*your expert statistical backed evidence doesn’t outweigh that in that sort of scenario*” [L622, Interview number and type withheld]. 

Interviewees felt that representations from members of the public could be more powerful than statistics, as board members were “*more interested in the anecdotal size of the problem*” [L349, Interview 1, ADP]. Others reported that board members were unable to relate to the statistical data being presented by interviewees or just generally distrusted that kind of evidence.

“*You have no idea how [the licensing board] interpret data. They don’t understand data… I’m not saying they’re not clever, it’s just if you’re not used to thinking in an academic way, then you can’t look at it and say how this relates to you.*”

[L968, Interview 10, Public Health]

“*There’s a whole group of non-believers out there. I had someone from the Licensing Board say to me that he didn’t believe any statistics ever.*”

[L380, Interview number and type withheld]

In the quasi-judicial environment of the licensing board meetings, the public health expertise of interviewees did not have the same status as in a health service context.

“*I didn’t even have an obvious place to speak…it’s done in a very formalistic way, the Chair introduces a topic and then asks for comments on it and people put their hands up and me, as a supposed expert, simply have to put my hand up along with other Councillors who want to have their say.*

*And then if one of the Councillors says something which is just completely ridiculous, which I have to say they do on occasions, you know, in the NHS I would be saying ‘excuse me but actually, they perhaps weren’t aware that this was the situation that had happened’. But you can’t just do that in these environments. So you get left looking as though you don’t know what you’re talking about and somebody who’s got completely no idea about a topic comes across as equally valid in their opinions to you as an expert*”

[L585, Interview 12, Public Health]

### 3.3. Role Perceptions

Much of the reported work of interviewees focused on the collation of data to support the development of new licensing policies, and in particular to feed information into licensing board decisions on whether or not to declare part (or all) of their area to be overprovided with licensed premises. It was emphasised that this data gathering, analysis and communication required “*a lot of intensive work over a prolonged period*” and that collaboration with others including the police and health colleagues was important, often through small multi-agency working groups. Most participants prepared detailed reports for the licensing boards on alcohol-related harm in the board area, and presented the findings to the boards, or submitted them as part of consultations on licensing policy. 

There was a tension apparent in the interviews, however, in relation to whether these efforts aimed to help the licensing board understand local data—as a kind of neutral support, providing “*impartial*” advice—or used the data actively to influence them towards decisions felt to favour public health. This tension manifested itself in the extent to which those analysing overprovision data presented their results in terms of options for the licensing board to take action, recommendations for a particular course of action, or a mixture of the two. One interviewee described his role as being “*about providing the facts, what [the licensing board] do with it is up to them*” [L172, Interview 3] but acknowledged that “*in our report we did make recommendations*”*.* Some interviewees reported that licensing board members viewed them as having a particular agenda, which made it more difficult to exert influence. Others questioned the motives of licensing board members. As public health practitioners, it felt difficult to be neutral:

“*Obviously in health we’re very aware of a lot of the problems and we think this is serious and we would like to do something about it. So it’s difficult to be completely neutral.*”

[L989, Interview 12, Public Health]

Participants were uncertain about whether and how, they should seek to influence individual licensing board members. In one area, the interviewee had started to monitor licensing applications and objections, and the voting pattern of each individual councillor at each licensing board meeting, though it was unclear how the data were going to be used. One interviewee had been told that they were not allowed to engage licensing board members on a one to one basis, as it would be seen as “*lobbying*”, which was against their organisational policy. Another felt that they needed to do more of that:

“*Next time I think we need to get to licensing board members more directly and start to brief them a bit more about the problems. There are opportunities to do that and we probably didn’t do enough of that. They’re elected members so we can meet with them.*”

[L377, Interview 1, ADP]

Most participants felt that very public campaigning, or a combative “*them and us*” approach to the licensing board, could be counterproductive but recognised the role of “*softly, softly lobbying; winning hearts and minds*” [L965; Interview 10, Public Health].

“*I think lobbying of board members would generally be counter-productive… [It] tends not to be something that goes down well with councillors that sit on a regulatory body…councillors…have to be absolutely impartial. If the ADP [*Alcohol and Drug Partnership—a strategic multi-agency group in each local area*] were viewed as a lobbying group it would undermine their credibility and independence.*”

[L322, Interview 8, Key Informant]

“*We changed the tack—instead of fighting [*the licensing board*], we said ‘let us support you, let us work with you. We understand the anxieties you have around this whole issue [*of overprovision*].’*”

[L120, Interview 4, ADP]

Participants emphasised the importance of this softer approach, in particular the need to build relationships and credibility with the licensing board and administrative staff over time. This was felt to be best achieved by continually engaging with the licensing process, including responding to individual applications as they came in and being regularly present at licensing board meetings—to achieve a kind of “*drip, drip effect*” [L502, Interview 13, Public Health] and so the licensing board members “*know who you are*” [L761, Interview 9, Public Health].

“*They kind of get used to my face. I go to the board meetings to see how they’re getting on so they start to see me, that might be a good thing or a bad thing—oh no here she comes again, always banging her drum about alcohol. So I’m starting to build up a relationship with them as well. One board member came to me after the last meeting and asked me ‘how did you think that went? Did you think we made the right decision?’ That was quite nice, they are obviously beginning to build up some trust in me…*”

[L166, Interview number and type withheld]

“*We are developing a beautiful working relationship with the licensing board and that’s what we’re looking for, that’s what it needs to be.*”

[L403, Interview 4, ADP]

## 4. Discussion

Following the introduction of a public health objective for Scottish alcohol licensing, some local public health practitioners embraced new roles in engaging with licensing officials and local licensing boards. Their reports give a sense of evolving practice in which a variety of ways of working, and the limits of what is appropriate, are being explored. As this unfolded, practitioners encountered different values and beliefs from their own about alcohol and evidence more generally, and some adapted their approach in response. 

Interviewees’ reported early approaches to this agenda demonstrate a “*naïve rationalism*” [49,50], in which simply providing enough data about health harms to licensing policymakers was expected to result in a change in policy in favour of public health. Notwithstanding successes in some areas [20], many were surprised at how little impact health evidence had in the licensing arena. This illustrates the persistence, or perhaps re-emergence, of the idea of an ideal-type model of “*evidence-based policymaking*” [34] which proposes that there “*can and should be a direct and unproblematic link between ‘the evidence’ and policy decisions and outcomes*” [50] (p. 2). In contrast, evidence from policy theory suggests that policy is neither solely nor directly based on scientific evidence, nor should it be [50,51], and this study suggests that licensing policy, as a political process, is no different. 

The idea held by some interviewees that alcohol licensing could or should seek to reduce whole-population alcohol consumption, may represent an optimistic view of the evidence base [28,29], and is relatively new. Licensing has historically had a strategic focus on the public good but health considerations, and particularly long-term health, have had little traction in day to day licensing [34]. An earlier study of the Licensing (Scotland) Act 2005 found that the public health objective of licensing was not well understood and fit poorly within a paradigm traditionally focused on harms at the level of individual licensed premises rather than the population harms of concern to public health [41]. While national policy can introduce a public health objective into the “institution” of licensing, that policy is implemented by “*street-level bureaucrats*” [52] who are subject to a range of sometimes nonspecific requirements laid down by central government—the lack of a clear definition of the public health objective, and “overprovision” of licensed premises being examples of this. Local policymakers, therefore, exercise discretion “*to satisfy a proportion of central government objectives while preserving a sense of professional autonomy necessary to maintain morale*” [50]. Thus, national policy can be changed or derailed when implemented locally [34] with local policymaking taking account of multiple aspects not necessarily dictated by the “institution” (or legal provisions) including in this case, economic considerations, perceived public opinion and doubts about evidence [53]—all of which are commonplace and legitimate considerations for policymakers, which are not changed overnight by the introduction of legislation. 

As a function of local government, licensing involves very different “*cultures of evidence*” to those with which public health professionals may be familiar [34,54,55,56,57]. The value of high-level review evidence on health indicators is considerably lower, and may be perceived as having limited transferable value if generated in other countries or contexts [50]. Academic studies have less evidential value as a matter of law than material fact (McGowan, cited in [34]). This study supports the conclusion that scientific evidence is interpreted in light of well-established ideas, values and beliefs [58], and other forms of evidence, personal experience, local knowledge, individual stories also (legitimately) carry weight [51,59]. When there are multiple ways to understand a problem, and “evidence” is contested, persuasion and argument—how problems and solutions are framed by stakeholders and understood by policymakers—become central to the acceptance of the public health (or any) interpretation of the best way forward [49,50,51]. This takes time, perhaps decades, to achieve [60]. Greater awareness amongst public health practitioners of the fact that health is often one of several competing goals of policy may help them to understand that evidence presented within political discussions does not necessarily tell policymakers what to do.

In this study, public health practitioners felt that involving the public in providing evidence to the licensing board was persuasive and that building a shared understanding of alcohol problems with licensing actors was a core aspect of their work. Analysis of the translation into policy of evidence on health inequalities has drawn on actor-network theory and suggests that policy entrepreneurs who actively package the evidence are needed, but that their potential success may be limited by the political context [61]. Considering compelling approaches to persuasively argue for public health goals within the distinct political context of a licensing board, rather than assuming evidence of health benefits is sufficient, should be considered. The greater involvement of the public in local decision-making (such as through deliberative processes) may provide opportunities for doing just this [62], but further research is needed before their broader adoption could be recommended. 

In this study, interviewees generally agreed that only by building positive working relationship with licensing actors could they expect to make progress. Overall, there is a sense that this, and the other “lessons” reported by participants were, in some cases, hard gained, despite being unsurprising to those familiar with policymaking theory [50,51,63]. Whilst there are many different theories of policy-making, key tenets include the tendency of policymakers to act in accordance with their beliefs using heuristics; the need to engage in long-term strategy and build strategic alliances to influence policy and the importance of stories and framing of evidence [50,64,65]. That no interviewee referenced policy studies or theory in their accounts illustrates a failure in the system by which such evidence should be communicated to, or accessed by, those who might benefit from it. Public health actors might then have utilised these strategies earlier and perhaps been less surprised by the reactions of licensing actors. 

This evidence, and the experiences of these public health practitioners, also suggests that it would be naïve to think that adopting a neutral position, in which one simply provides facts/data to the licensing board, would be the most effective way to influence decisions towards public health. Furthermore a neutral position is neither desirable, nor possible. The choice of data, and framing of those data in written and verbal presentations to the licensing board cannot be value-free. Public health actors are expected, and indeed paid, to act to maximise the public health of the communities they serve, provided they do so within appropriate ethical frameworks [66,67,68]. The nature of public health involvement in licensing, therefore, is, and ought to be, one of advocacy—aiming to build relationships with allies and licensing actors, and be useful to those actors—while engaging in gentle attempts to make health considerations part of the routine practice of licensing [34]. This is a form of lobbying; not the overt or loud campaigning the term may bring to mind for some, but similar to that undertaken by the alcohol industry with the United Kingdom government over many years [69,70,71], seemingly to considerable effect [72], though with markedly different goals.

Further research in this field could examine the potential influence of greater public involvement on licensing decisions, as well as developing a clear consensus on potential mechanisms of effect of licensing in terms of benefits such as reduced alcohol-related harms [73], and how public health involvement might influence the outcomes. There is clearly a need for empirical testing of the impact of overprovision policies, public health involvement in licensing more broadly and any specific impact attributable to the introduction or continued existence of a public health objective for licensing. 

### Strengths and Limitations

This is the first study to specifically focus on the perspective of public health practitioners in the licensing system: it brings that perspective into focus and highlights some of the challenges that remain in establishing public health as a consideration in alcohol retail regulation. Interviews were in-depth and the sample included experienced representatives from almost all areas in Scotland where public health actors had been actively engaging with the licensing process. Participants, mostly suggested by Alcohol Focus Scotland (AFS), included practitioners from a range of backgrounds including doctors within local health boards’ public health teams, and local Alcohol and Drug Partnership staff based within local authorities or health boards. While there is no reason to doubt the veracity of their reports, they are grounded within a specific perspective which may converge towards support for a whole population approach to alcohol policy, advocated by AFS. Their reports necessarily reflect the Scottish licensing context, but highlight issues relating to values, cultures of evidence, and local policymaking which can inform debates and theories elsewhere. As the findings align well with established policy theory more generally, it is also likely that they may inform other efforts to achieve structural change in the local environment for public health benefit. 

## 5. Conclusions

Public health practitioners experienced a degree of disappointment that the introduction of the public health objective to Scottish alcohol licensing did not quickly result in a transformation in the goals and decisions of local alcohol licensing officials. Alcohol licensing is represented as a political process in which decision-making is influenced by prior values and beliefs and consideration of the needs of a broad range of stakeholders, and evidence over and above scientific data. Public health will take time to become established as a routine consideration within that environment. Public health practitioners may learn from policy theory, as their experiences reported here are strikingly in accord with such theory. Efforts to shape ideas and beliefs about alcohol and the role of licensing, through relationship-building with licensing actors over a prolonged period, seem vital to making progress going forward. 

## Figures and Tables

**Table 1:** Licensing objectives (current or proposed in the UK).

Licensing (Scotland) Act 2005 Objectives	Licensing Act 2003 (England and Wales)	Northern Ireland (Previously Proposed)
For the purposes of this Act, the licensing objectives are— (a) preventing crime and disorder, (b) securing public safety, (c) preventing public nuisance, (d) protecting and improving public health, and (e) protecting children and young people from harm.	The licensing objectives are— (a) the prevention of crime and disorder; (b) public safety; (c) the prevention of public nuisance; and (d) the protection of children from harm.	(a) Promotion of public health. (b) Promotion of public safety. (c) Prevention of crime and disorder. (d) Prevention of public nuisance. (e) Protection of children from harm. (f) Fair treatment of all stakeholders.

**Table 2:** Profile of interviewees (*n* = 13).

Descriptor	Breakdown ^a^
Organisation (number of interviewees)/Role:	Alcohol and Drug Partnership (ADP) (6)/various roles including ADP co-ordinator and more junior officers of the ADP. National Health Service (NHS)/Public Health (5)/public health consultants, trainee consultants, or junior doctors. Key Informants (2): Alcohol Focus Scotland, a third sector organisation working to reduce alcohol-related harm (1); Recognised local licensing expert (1)
Health Board areas in which interviewees had experience:	Eight of 14 health board areas were covered.
Licensing Board areas in which interviewees had experience:	Twenty of 40 licensing boards were covered.
Licensing Board areas policy status:	The extent to which the sample included licensing boards which had declared overprovision (OP) was analysed using data on policies published by 30 April 2014 [44]: Widescale OP declared (4) Limited OP declared (4) No OP declared (5) Policy not published (7) Of the 20 licensing board areas not included in the sample, 18 had not declared OP, 1 had declared limited OP, and 1 had declared widespread OP.

^a^ An individual breakdown is not provided to protect participant anonymity.

**Table 3:** Main questions in interview topic guide.

Main Questions in Interview Topic Guide
How did you get involved with the issue of overprovision of licensed premises (in your area/organisation)? Who else was involved in the initiative? How were they involved? How did you build support/win “hearts and minds” with different stakeholders? When and how were community members/the general public involved in the initiative? What data did you collect and why? How successful do you think your efforts have been? What else can/should be done locally on this agenda? What would you do differently if starting this process? What else can be done nationally on this agenda? From all that you’ve mentioned, what would you pick out as the key lessons for others trying to take action on identifying and addressing overprovision in their area?

**Table 4:** Overall analysis framework.

**1. Learning, Expertise, Capacity, Persistence**	
1a. Learning about Influencing Licensing	*Formal and informal mechanisms of learning for* *public health (PH) actors—peer support, national guidance.*
1b. Other Expertise	*Data analysis, legal and economic expertise—confidence and availability of expertise*
1c. Long term approach, persistence	*Timing, preparation, planning for future, taking a long-term view. Reviewing all licensing (L) applications;*
1d. Capacity	*Capacity to respond regularly and rapidly; Level of effort/time spent/required.*
**2. Working with Others**	
2a. Alliances	*PH actors from various organisations working in partnership with public sector colleagues from other organisations on licensing issues*
2b. PH Actors working with Licensing Actors	*Perceptions and reports of working with licensing standards officers, L clerks, L boards. Mechanisms for communication with L actors.*
2c. Helping or Influencing	*Efforts to influence licensing board (LB) members and how such efforts are framed/perceived—helping* versus *lobbying/campaigning. Presentation of “recommendations” or “options” to LBs*
2d. Raising awareness	*Efforts to inform LB and other stakeholders about alcohol harm, overprovision* etc.
2e. Building relationships	*Relationship building with LB and others; time needed to build; more than awareness—“hearts and minds*”
**3. Power, Autonomy, Bias**	
3a. Licensing board autonomy & accountability	*Independence and control of LBs. Mechanisms to hold LBs accountable for upholding the L Scotland Act or implementation of L objectives.*
3b. Legalistic licensing system	*Formal and legal processes and requirements; Disempowerment of LB outsiders; disempowerment of LB—fear of litigation.*
3c. Conflicts of interest (COIs)	*Ability of individuals and organisations to act independently and without bias—for PH actors and others. Types of bias—host organisation; personal interests…Issues about representation on forums are not included here, but in 6a, 6a, though COIs of individuals on forums would be included.*
3d. Power and influence of individuals	*The influence of individuals on action and progress. Lack of continuity when personnel/LB membership changes.*
**4. Evidence**	
4a. Defining overprovision of L premises	*Challenges and difficulties in defining overprovision (OP); choices re. geographical unit of analysis; historical practices and understanding of OP*
4b. Hard (imperfect) data	*Emphasis on quantitative data, challenges of measuring capacity and provision, relating harm to provision.*
4c. Presentation of evidence	*Oral and written presentations; importance of presenter, and clarity and simplicity of data presented.*
4d. Softer data	*Importance and power of qualitative evidence and public opinion—anecdote/personal experience; (Methods used to collect data are covered in 6 d)*
4e. Perceptions of data	*Ownership of evidence; acceptance of evidence; attitudes towards harder and softer evidence including public views.*
**5. Attitudes and Beliefs Regarding Alcohol and Alcohol Licensing**	
5a. Attitudes to alcohol in general	*Perceptions of alcohol problems; sense of problems only in other places or groups;*
5b. Role of licensing in relation to PH & other objectives	*Importance of mood of L board; focus on short term issues (e.g., disorder) or long term (e.g. health); acceptance of availability as driver of consumption and harm;*
5c. Views on the Effectiveness of the L system to address alcohol-related harm.	*Perceived limitations of the L system in improving public health. Return on time invested in taking action on this issue. L as just part of a larger alcohol policy picture.*
5d. Economic arguments	*How economic issues influence licensing decisions; lack of data/method to compare risks/benefits of new L applications; beliefs in economics being more important than PH*
**6. Public and Stakeholder Involvement**	
6a. Forum as Public Involvement mechanism	*Representativeness of members; appointment of members.*
6b. Functioning of forums	*Effective operation; representation of stakeholder views to the LB: conflicts within forums*
6c. LB statutory consultation	*Breadth of formal consultation; scope; standards; impact*
6d. PH-led consultation/research into public views	*Methods used; questions asked; groups and numbers involved; impact*
6e. PH-led public engagement/empowerment	*Engagement; awareness raising; support; empowerment; campaigning. Public power.*
